# An Automated Analysis Tool for Diffusion Tensor Imaging‐Based Quantitative MRI in X‐Linked Adrenoleukodystrophy

**DOI:** 10.1002/jimd.70108

**Published:** 2025-10-26

**Authors:** Eda G. Kabak, Fenna van Dodewaard, Stefan D. Roosendaal, Hemmo A. F. Yska, Marije M. C. Voermans, Marine Bourgeais, Vincent Perlbarg, Marc Engelen

**Affiliations:** ^1^ Department of Neurology and Pediatric Neurology, Emma Children's Hospital, Amsterdam Leukodystrophy Center Amsterdam University Medical Center Amsterdam AZ the Netherlands; ^2^ Faculty of Medicine University of Amsterdam, Amsterdam University Medical Center Amsterdam AZ the Netherlands; ^3^ Department of Radiology Amsterdam UMC Location University of Amsterdam Amsterdam AZ the Netherlands; ^4^ BrainTale SAS Paris France

**Keywords:** brainQuant, cerebral ALD, diffusion tensor imaging, myelopathy, quantitative MRI, radial diffusivity, X‐linked adrenoleukodystrophy

## Abstract

X‐linked adrenoleukodystrophy (X‐ALD) is caused by *ABCD1* pathogenic variants, leading to accumulation of very long‐chain fatty acids (VLCFAs). Phenotypes include cerebral ALD (CALD) and adrenomyeloneuropathy (AMN). We assessed if quantitative MRI (qMRI) parameters from an automated tool (BrainQuant) could differentiate CALD from non‐CALD and reflects myelopathy severity. Adult males from a prospective study (2015–2024) underwent annual neurological exams and brain MRI. Exclusion criteria were: fewer than two MRIs, non‐ALD lesions, or prior transplantation. BrainQuant processed diffusion tensor imaging (DTI) to yield global metrics: fractional anisotropy (FA), mean diffusivity (MD), axial diffusivity (AD), and radial diffusivity (RD). Patients were stratified by Loes score (0 vs. > 0) and expanded disability status scale (EDSS ≤ 2 vs. > 2). Longitudinal changes were modeled. Correlations with EDSS, severity score for progressive myelopathy (SSPROM), and 6‐min walk test (6‐MWT) were analyzed. The cohort included 62 patients (median age 36.5); 15 had CALD. Global DTI metrics did not differ significantly between CALD and non‐CALD. A trend for higher radial diffusivity (RD) in the splenium (*p* = 0.037) was seen, but results were not significant after Bonferroni correction for multiple comparisons (*p* = 0.748). Patients with EDSS > 2 showed significantly worse global DTI values (*p* < 0.05), correlating with clinical scores (*r* = 0.40–0.69). Longitudinally, RD‐global increased significantly (*p* < 0.001) at similar rates across EDSS groups. BrainQuant qMRI did not distinguish CALD and non‐CALD but effectively tracked myelopathy. Radial diffusivity (RD)‐global is a promising biomarker for monitoring X‐ALD progression.

## 1 Introduction

X‐linked adrenoleukodystrophy (X‐ALD) is a progressive neurometabolic disorder caused by pathogenic variations in the ABCD1 gene, affecting the ABCD1 protein. This genetic defect results in impaired peroxisomal beta‐oxidation of very long‐chain fatty acids (VLCFAs), leading to accumulation in all cells in the body. However, only specific organs are affected, primarily the adrenal cortex and central and peripheral nervous systems. The clinical manifestations are highly heterogeneous, ranging from adrenal insufficiency and slowly progressive myelopathy to leukodystrophy. The leukodystrophy (cerebral ALD; CALD) primarily affects young boys and results in significant cognitive and neurological impairments [1, 2].

In patients with pathogenic variants, leukodystrophy has a lifetime prevalence of 40%–60% [3]. Lesions typically start in the splenium of the corpus callosum. Other commonly affected anatomical regions include the corticospinal tracts, internal capsule, and lemniscus medialis. In children, 80%–85% of cerebral lesions are progressive, while 10%–15% exhibit spontaneous arrest of the disease for many years [4, 5].

Spinal cord involvement develops in virtually all male patients and more than 90% of female patients by the age of 60 [6]. The dorsal columns and pyramidal tracts of the spinal cord are affected, resulting in spastic paraparesis, sensory ataxia, and bladder dysfunction [7, 8, 9].

Early diagnosis in male patients has benefits because monitoring allows for the early identification of adrenal failure (treatable with hormone supplementation therapy) and CALD (treatable with allogeneic hematopoietic cell transplant in patients with early disease). This is the rationale for including ALD in newborns' screening programs [10]. Monitoring of disease includes adrenal function testing and MRI scans of the brain at regular intervals [1]. Currently, the detection of leukodystrophy relies on conventional MR imaging and scoring affected brain regions with the Loes score [11], which may overlook subtle changes in the white matter. Previous studies have demonstrated that lesion progression, as scored by the current system, is underestimated compared to lesion volumetrics [4]. Other quantitative MRI (qMRI) parameters such as DTI metrics with Fractional Anisotropy (FA), Mean Diffusivity (MD), Axial diffusivity, and Radial Diffusivity (RD) have shown sensitivity to lesion progression and clinical severity, even in early stages in boys [12, 13]. Therefore, qMRI parameters may be promising alternatives in the early detection of disease onset and follow‐up.

The spinal cord disease of ALD is slowly progressive over time, with changes detectable after 2 years of follow‐up with conventional clinical outcome measures [8, 9, 14]. Surrogate outcome measures are needed for clinical trials. Conventional imaging of the spinal cord shows atrophy, but only in advanced disease over very long periods of time [13].

Quantitative magnetic resonance imaging (qMRI) of the motor tracts shows promise as a surrogate outcome [15, 16], but implementation has been limited by larger inter‐center/inter‐scanner measurement variability. The brainQuant module, which enables generalizing of qMRI parameters, has been developed with the aim to reduce this inter‐scanner variability, allowing analyses of multicentric data and has been recently used to monitor disease progression in a small cohort of CALD patients [17].

The “Dutch ALD cohort” is a large prospective natural history study for ALD. We used data from this cohort to study if the analysis of MRI scans with brainQuant could (1) show differences between patients with and without leukodystrophy (CALD) and (2) correlate with the severity of spinal cord disease and change over time.

## 2 Methods

### 2.1 Study Design and Participants

Male patients with a pathogenic variant in the ABCD1 gene and biochemical confirmation with elevated VLCFA levels were prospectively recruited at the AUMC between 2015 and 2024 as part of the “Dutch ALD cohort” [8]. All men, including children, are included in the “Dutch ALD cohort.”

For the current analyses, only men with a minimum age of 18 years were included. All patients were assessed yearly, including standardized neurologic examination and MR of the brain. The presence of cerebral white matter lesions was evaluated by examination of T2‐weighted scans by experienced neuroradiologists, and all scans were evaluated using the Loes score by one neuroradiologist to decrease intra‐rater variability [SR].

Patients with white matter abnormalities not caused by ALD and with a history of hematopoietic stem cell transplantation (HSCT), gene therapy, or compassionate use of leriglitazone were excluded. Patients with less than two consecutive MRI scans were excluded from the longitudinal analyses.

Disease severity was quantified by several functional outcomes such as the 6‐min walk test (6‐MWT) [8, 18, 19]. Clinical outcome was assessed by the expanded disability status scale (EDSS), severity score for progressive myelopathy (SSPROM), as described earlier in this cohort [8, 18, 19].

For cross‐sectional analyses, patients were stratified by a Loes score (either 0 or > 0). A Loes score of zero indicates the absence of ALD‐related cerebral white matter lesions, whereas a score of > 0 indicates the presence of lesions, meaning patients who were scored as having CALD had a Loes score > 0. Patients were also stratified for clinical severity using an EDSS score of 2.0 as a cut‐off, as a score > 2.0 represents more than mild disability.

Sixteen male healthy controls were also included. The requirements for control patient eligibility were a minimum age of 18 years and absence of neurologic comorbidity.

### 2.2 Ethics and Consent

This study was conducted in accordance with the Declaration of Helsinki (1975, revised 2000). The study protocol was approved by the Medical Ethics Committee of Amsterdam UMC (METC #2018_310, approved 2018). Written informed consent was obtained from all participants or their legal representatives. The privacy rights of human subjects were observed. No organs or tissues were used in this study.

### 2.3 MRI Acquisition

Imaging of the cerebrum was performed on two different 3‐Tesla MR scanners (Ingenia Elition X and Ingenia, Philips Medical Systems, Netherlands) using a 32‐channel head coil. Acquired sequences included 3DT1 and 3D fluid attenuated inversion recovery (FLAIR) at 0.9–1.00 mm isotropic resolution and 2D axial T2. One multi‐shell DTI sequence (with *b* values 0, 1000, and 2000 s/mm^2^) with single spin‐echo echo‐planar images was obtained and acquired at 2.5 mm isotropic resolution. The single‐shell data were used to extract classic DTI measures. The detailed acquisition measurements of DTI included repetition time (TR) ranging from 2290 ms to 7710 ms, echo time (TE) of 95 ms, voxel size 2.5 × 2.5 × 2.5 mm, slice thickness 3 mm, and 1.5× SENSE acceleration. Acquisition time was around 40 min. Further details of the used protocol have been extensively described earlier by Stellingwerff et al. [20].

#### 2.3.1 Post Processing Using brainQuant

DICOM data were directly uploaded to the brainTale‐care platform version 6.0 (CE marked, BrainTale, Paris, France) and were post processed with the brainQuant module (version 5.0) dedicated to the extraction of reliable quantitative diffusion tensor imaging (DTI) markers, in controlling inter‐scanner and inter‐protocol variability by implementing a calibration procedure.

#### 2.3.2 BrainQuant Module

The used calibration procedure is based on establishing a set of reference normal values based on healthy control subjects for each MR‐DTI protocol used on a given scanner. Subsequently, all measures obtained from patients with the same protocol are expressed relatively to these reference values. The required acquisition parameters for the brainQuant module are based on studies by Van der Eerden et al. [21] and Puybasset et al. [22], and include a minimal voxel size of 3 × 3 × 3 mm, with a minimal *b* value of 1000 mT/m and measurements along at least 12 gradient directions [21].

The processing pipeline corrects for movement artefact using a linear co‐registration method via NiftyReg (King's College, London, UK). Brain masks are estimated using an AI‐driven method and applied to *b* = 0 s/mm^2^ images after validation against FLIRT, FSL (FMRIB Software Library) [23]. Diffusion Imaging in Python (Dipy) is employed for tensor model fitting via the recons.dti and dipy.reconst.dti modules and the instances TensorModel and TensorFit to obtain quantitative measures [24].

#### 2.3.3 ROI Selection

**FIGURE 1:**
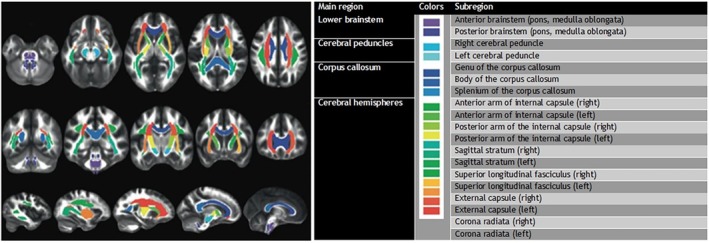
Atlas of cerebral white matter regions used for DTI analysis, showing 19 regions of interest in MNI space.

The maps of DTI parameters were co‐registered to the standard Montreal Neurological Institute (MNI) space using a non‐linear method in NiftyReg. Mean values for these parameters were then extracted from 19 pre‐defined deep white matter regions of interest (ROIs), which were selected from the Mori et al. white matter atlas [25]. For the analysis of cerebral ALD (CALD), these ROIs covered a broad range of anatomic structures (Figure 1). For spinal cord disease, the analysis focused specifically on ROIs related to the corticospinal tracts, including the cerebral peduncles, internal capsule, and brainstem.

#### 2.3.4 Data Calibration

To reduce inter‐scanner variability, the data is then calibrated by calculating the mean values of the quantitative measures for healthy controls. A minimum of five healthy volunteers is needed per MRI protocol. Calibrated data are then calculated by dividing patients' values by the values of the healthy controls of the same ROIs obtained from the same scanner and the same MRI protocol.

The healthy volunteers underwent the exact same MRI protocol as the patient cohort.

To account for inter‐individual variability, the standard deviation (SD) of each calibrated value is calculated from a previously acquired cohort (NCT00577954) [22] and the results are expressed as the number of SDs from the mean of healthy volunteers.

Each MRI examination and the postprocessing steps are checked for motion, signal drop‐out, number of global signal dropouts, vibration artefacts, and visual inspection of FA map co‐registration in MNI space.

#### 2.3.5 Quantitative Measures

Within the brainQuant module, only *b* = 0 s/mm^2^ and *b* = 1000 s/mm^2^ were used to estimate the tensor module to be consistent with the reference data which was validated, as described in Section 2.3.2. Subsequently, the brainQuant module was used to obtain fractional anisotropy (FA), mean diffusivity (MD), axial diffusivity (AD), and radial diffusivity (RD). Also, FA‐global, MD‐global, AD‐global, and RD‐global, which are the average values of the quantitative measures in the previously described 19 regions, were obtained. The global values are expressed as percentages or as numbers of SD in normal controls.

### 2.4 Statistical Analyses

Statistical analyses were performed using IBM SPSS Statistics version 28.0 (Armonk, NY: IBM Corp) and RStudio version 4.3.2 (Posit PBC, Vienna, Austria).

Baseline data was summarized using means with SDs (normally distributed data) or medians with interquartile ranges (IQR) for non‐normally distributed data. The normality of data was assessed by the Shapiro–Wilk and Kolmogorov‐Smirnov tests for normality.

The data was first analyzed cross‐sectionally at baseline level. The patient cohort was stratified for EDSS ≤ 2.0 and EDSS > 2.0 and the presence or absence of cerebral ALD. Differences in means for DTI parameters RD‐global, AD‐global, MD‐global, and FA‐global were assessed using the Mann–Whitney U test for non‐normally distributed data and the *t* test for normally distributed data.

For the DTI parameters that differed significantly between groups, correlations between clinical outcome measures and DTI parameters were assessed using Pearson correlation (normally distributed continuous data) or Spearman rank‐order correlation (non‐normally distributed continuous data). We considered a correlation of > 0.30 clinically relevant. A correlation between 0.30–0.39 was accepted as a moderate relationship, 0.40–0.69 as a strong relationship, and > 0.70 as a very strong relationship [26].

As we hypothesized that the RD would be most specific for myelin dysfunction, we analyzed the between group differences for RD in the separate anatomic regions, and we subsequently evaluated correlations between the RD of the previously described ROIs and clinical parameters using either Pearson correlation or Spearman rank‐order correlation.

To assess longitudinal changes for the DTI parameters that differed significantly between separate groups and were correlated with clinical parameters, we employed mixed linear models (non‐normally distributed data). Estimated marginal means (EMMs) were calculated. Visit number, EDSS subgroup, and CALD presence were included as fixed effects, adjusting for age at baseline. We accounted for inter‐individual variability using a random intercept for each participant. We employed a group‐by‐time interaction in the linear mixed model to evaluate longitudinal group differences. Post hoc pairwise comparisons were performed to explore differences between visits.

Effect sizes for change were reported as generalized eta squared (*η* ^2^G), with 0.001–0.006 small effect, 0.3–0.5 medium effect, and > 0.5 large effect.

Results were corrected for multiple comparisons according to Bonferroni. Estimated means, standard errors, and *p* values after correction are presented. *p* values lower than 0.05 were considered statistically significant.

## 3 Results

### 3.1 Participants

**FIGURE 2:**
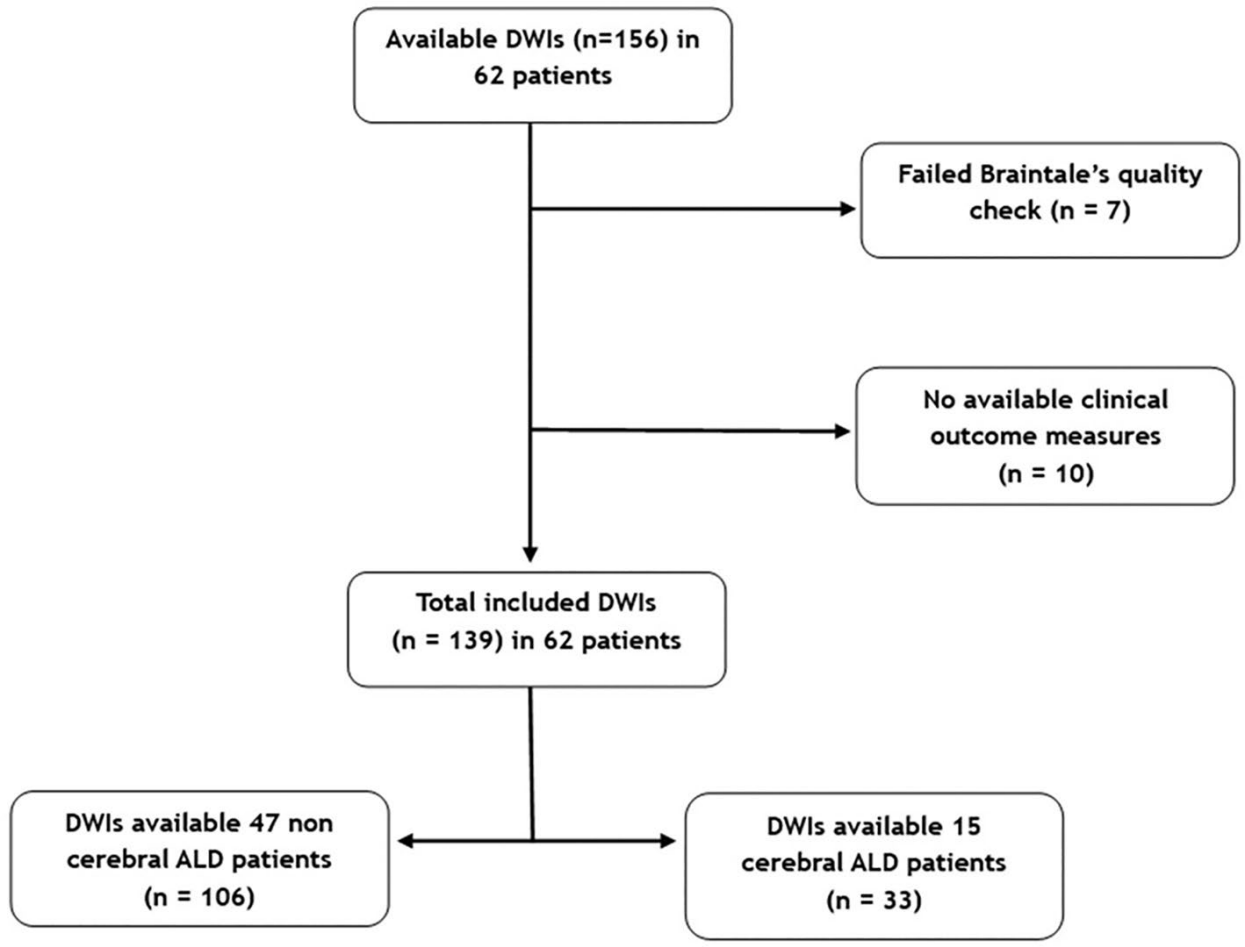
Flowchart of the diffusion‐tensor imaging (DTI) selection process for the study cohort. DTI, diffusion tensor imaging.

For the baseline analyses, 62 patients were included. A total of 139 suitable DW‐imaging scans were retrieved for 62 patients who were followed yearly. A detailed description of the brain imaging selection process is provided in Figure 2.

The median age of the cohort was 36.5 years. Fifteen patients presented with CALD lesions at the first baseline visit. Of the entire cohort, 29% used an assistive walking device, while 3.2% were dependent on a wheelchair. Baseline characteristics for the cohort are summarized in Table 1.

**TABLE 1:** Baseline characteristics of the X‐ALD cohort, stratified by CALD and EDSS groups, including SSPROM, 6‐MWT, and SF‐36 outcomes.

Characteristics	All patients (*n* = 62)	EDSS ≤ 2 (*n* = 24)	EDSS > 2 (*n* = 38)	*p* *	No cALD (*n* = 47)	cALD (*n* = 15)	*p* **
Age	36.50 (28.75–59.25)	26.50 (21.00–34.50)	52.50 (35.00–62.50)	< 0.001	37.00 (28.0–59.0)	35.00 (32.00–60.00)	0.856
SSPROM	86.75 (76.00)	100.00 (98.00–100.00)	78.00 (71.50–86.13)	< 0.001	87.00 (77.50–100.0)	82.50 (72.00–100.00)	0.568
6‐MWT	510.33 (±178.17)	657.26 (±91.93)	401.32 (±145.10)	< 0.001	526.20 (±168.12)	465.00 (±204.07)	0.348
SF‐36							
Physical functioning	72.50 (31.25–100.00)	100.00 (95.00–100.00)	45.00 (20.0–70.00)	< 0.001	95.00 (27.50–100.0)	67.50 (30.00–100.00)	0.489
Physical health	100.00 (25.00–100.00)	100.00 (100.00–100.00)	50.00 (0.00–100.00)	< 0.001	100.00 (75.0–100.0)	100.00 (25.00–100.00)	0.259
Emotional problems	100.00 (72.03–100.00)	100.00 (100.00–100.00)	100.00 (66.67–100.00)	0.260	100.00 (100.0–100.0)	100.00 (66.69–100.00)	0.526
Energy/fatigue	50.00 (45.00–63.75)	55.00 (50.00–78.75)	47.50 (40.00–55.00)	< 0.001	55.00 (40.0–65.0)	50.00 (45.00–62.50)	0.964
Emotional well‐being	44.00 (36.00–75.00)	70.00 (40.00–87.00)	38.80 (33.00–44.00)	< 0.001	40.00 (36.0–44.0)	44.00 (36.00–76.00)	0.274
Social functioning	50.00 (42.50–76.25)	77.50 (50.0–100.00)	45.00 (37.50–60.25)	0.002	57.50 (30.0–100.0)	50.00 (42.50–75.00)	0.822
Pain	80.00 (67.50–100.00)	100.00 (82.0–100.00)	67.50 (45.00–90.00)	< 0.001	90.00 (75.13–100.0)	77.50 (42.50–75.00)	0.164
General health	55.00 (50.00–65.00)	60.00 (51.25–70.00)	55.00 (50.00–63.75)	0.086	55.00 (50.0–60.0)	60.00 (50.00–65.00)	0.556
Degree of disability							
Independent	44.00 (71.00)	24.00 (100)	20.00 (52.60)	< 0.001	34.00 (72.30)	10.00 (66.70)	0.676
Use of assistive walking device	18.00 (29.00)	0.00 (0)	18.00 (47.40)	< 0.001	13.00 (27.70)	5.00 (33.30)	0.676
Wheelchair dependent	2.00 (3.20)	0.00 (0)	2.00 (5.30)	0.257	1.00 (2.10)	1.00 (6.70)	0.390
Hormonal substitution							
Glucocorticoid use	35.00 (56.50)	16.00 (66.7)	19.00 (50.0)	0.201	24.00 (51.10)	11.00 (73.30)	0.133
Mineralocorticoid use	10.00 (16.10)	3.00 (12.5)	7.00 (18.40)	0.540	4.00 (8.50)	6.00 (40.00)	0.004
Tripping	23.00 (37.10)	2.00 (8.3)	21.00 (55.30)	< 0.001	17.00 (36.20)	6.0 (40.00)	0.895

*Note:* Data is presented as either mean (SD), median (IQR) or number of patients and percentages.

Abbreviations: 6‐MWT, 6‐min walking test; cALD, cerebral adrenoleukodystrophy; EDSS, expanded disability status scale; IQR, inter quartile range; SD, standard deviation; SF‐36, short form health survey; SSPROM, severity score for progressive myelopathy.

* *p* value group differences EDSS ≤ 2 and EDSS > 2.

** *p* value no CALD and CALD patients.

When stratified by the presence of CALD lesions, there were no statistically significant differences in clinical characteristics, with the exception of mineralocorticoid use. However, when patients were stratified according to EDSS severity, significant differences were observed in the outcomes of the 6‐Minute Walk Test (6‐MWT) and various quality of life domains. A detailed overview of these results by group is presented in Table 1.

Follow‐up data were available for up to 6 years for a subset of patients. To account for missing data due to loss to follow‐up, a complete case analysis was performed.

### 3.2 Between Group Differences

#### 3.2.1 CALD vs. Non‐CALD

We assessed differences in the mean ranks of DTI parameters FA‐global, MD‐global, AD‐global, and RD‐global across the nineteen regions previously defined (Figure 1) for patients with (*n* = 15) and without a positive Loes score (i.e., > 0) (*n* = 45). Mean ranks for MD‐, AD‐, and RD‐global (Figure S1) were higher in patients with a positive Loes score, whilst lower for FA‐global. However, no statistically significant differences were observed (Table 2). We then evaluated differences in the mean rank of RD for each individual region. In this cohort, regions frequently identified as affected by the Loes score included various parts of the brainstem (*n* = 7), the corpus callosum (*n* = 8), and the internal capsule (*n* = 8).

**TABLE 2:** Baseline differences in qMRI parameters (RD, AD, MD, FA) between CALD and non‐CALD patients.

qMRI parameters	No cALD	cALD	*p* *
RD‐global	30.19	35.60	0.320
AD‐global	30.65	34.13	0.525
MD‐global	30.26	35.40	0.344
FA‐global	32.64	27.93	0.387

Abbreviations: AD, axial diffusivity; cALD, cerebral adrenoleukodystrophy; FA, fractional anisotropy; MD, mean diffusivity; *p* value: Bonferroni adjusted significance; qMRI, quantitative MRI; RD, radial diffusivity.

* Bonfferoni adjusted significance of *p* values obtained by the Mann–Whitney U test.

Apart from the splenium of the corpus callosum, no significant differences in RD were found in other brain regions (*p* = 0.037), with CALD patients having a higher RD. However, no statistically significant differences between patients with and without white matter lesions were seen after post hoc correction (*p* = 0.748). See Table S1 for all detailed results.

#### 3.2.2 Spinal Cord Disease: EDSS ≤ 2.0 and EDSS > 2

Differences in mean ranks for all DTI parameters across the total nineteen regions were assessed; RD, MD, AD, and FA global differed significantly between the two groups (Table 3 and Figure S2). For the individual regions, RD also differed statistically significantly, being higher in the EDSS > 2.0 group.

**TABLE 3:** Baseline differences in qMRI parameters (RD, AD, MD, FA) between EDSS ≤ 2 and EDSS > 2 groups.

qMRI parameters	EDSS ≤ 2.0	EDSS > 2	*p* *	adjusted *p* **
RD‐global	19.75	38.92	< 0.001	< 0.001
AD‐global	22.67	37.08	< 0.001	0.002
MD‐global	20.37	38.53	< 0.001	< 0.001
FA‐global	41.04	25.47	< 0.001	< 0.001

Abbreviations: AD, axial diffusivity; cALD, cerebral adrenoleukodystrophy; FA, fractional anisotropy; MD, mean diffusivity; qMRI, quantitative MRI; RD, radial diffusivity.

* Mann–Whitney U test.

** Bonferroni adjusted significance.

RD‐global, MD‐global, and AD‐global were shown to be higher in patients with an EDSS above 2.0, whilst FA‐global was lower (Table S2).

Significant differences were also seen after post hoc pairwise comparisons between EDSS **≤** 2.0 (*n* = 24) and EDSS > 2 (*n* = 38) for both FA, AD, MD, and RD global. When looking at the individual regions, RD remained significantly different between the two groups in the cerebral peduncles, the external capsule, the corona radiata, and the left posterior limb of the internal capsule (PLIC).

#### 3.2.3 Correlation DTI Parameters and Clinical Outcome Measures

Strong, statistically significant correlations (correlation coefficient 0.40–0.69) were observed between clinical outcome measures and nearly all DTI parameters across the entire cohort (Table 4). The correlations between the AD global and both the EDSS and SSPROM were weak (correlation coefficient 0.10–0.30).

**TABLE 4:** Correlation coefficients between qMRI parameters and clinical outcomes (EDSS, SSPROM, 6‐MWT) across the cohort.

RD‐global Spearman's rho	EDSS	SSPROM	6‐MWT
Correlation coefficient	0.51	−0.57	−0.54
*p*	< 0.001	< 0.001	< 0.001
*N*	62	62	54

MD‐global Spearman's rho	EDSS	SSPROM	6‐MWT
Correlation coefficient	0.48	−0.52	−0.52
*p*	< 0.001	< 0.001	< 0.001
*N*	62	62	54

AD‐global Spearman's rho	EDSS	SSPROM	6‐MWT
Correlation coefficient	0.38	−0.40	−0.44
*p*	0.003	< 0.001	< 0.001
*N*	62	62	54

FA‐global Spearman's rho	EDSS	SSPROM	6‐MWT
Correlation coefficient	−0.46	0.53	0.49
*p*	< 0.001	< 0.001	< 0.001
*N*	62	62	54

*Note:* *p* value after Bonferroni correction for multiple comparisons.

Abbreviations: 6‐MWT, 6‐min walking test; AD, axial diffusivity; cALD, cerebral adrenoleukodystrophy; EDSS, expanded disability status scale; FA, fractional anisotropy; MD, mean diffusivity; qMRI, quantitative MRI; RD, radial diffusivity; SSPROM, severity score system for progressive myelopathy.

Among all clinical outcome measures, the strongest correlations were seen with the RD global (Figure S3).

For FA, global negative correlations were seen with the EDSS and a positive correlation with the SSPROM and 6‐MWT, indicating a reduction in FA global with disease progression.

In contrast, the other three DTI markers showed positive correlations with the EDSS and negative correlations with the SSPROM and 6‐MWT, reflecting an increase in these markers with an increase in disease severity.

Focusing on the RD in specific brain regions, significant strong correlations were observed for all clinical outcome measures with RD in the genu and body of the corpus callosum. Significant moderate correlations with the 6‐MWT were also found with RD in the internal capsule, including the anterior and posterior arms, bilaterally. All other correlations were statistically significant but weak to moderate (Table S3).

### 3.3 Longitudinal Analyses

A total of 25 patients (40%) had Diffusion Tensor Imaging (DTI) data available for the two‐year follow‐up assessment following their baseline evaluation.

Fifteen patients had an EDSS of 2.0 or below, whereas 10 patients had an EDSS above 2.0.

#### 3.3.1 DTI Parameter Changes Over Time: EDSS ≤ 2.0 and EDSS > 2.0

Linear mixed models were used to assess longitudinal changes in RD, AD, MD, and FA between baseline (V0) and follow‐up visits (V1, V2) in the EDSS subgroups. The mean time between visits was 11.8 months.

**FIGURE 3:**
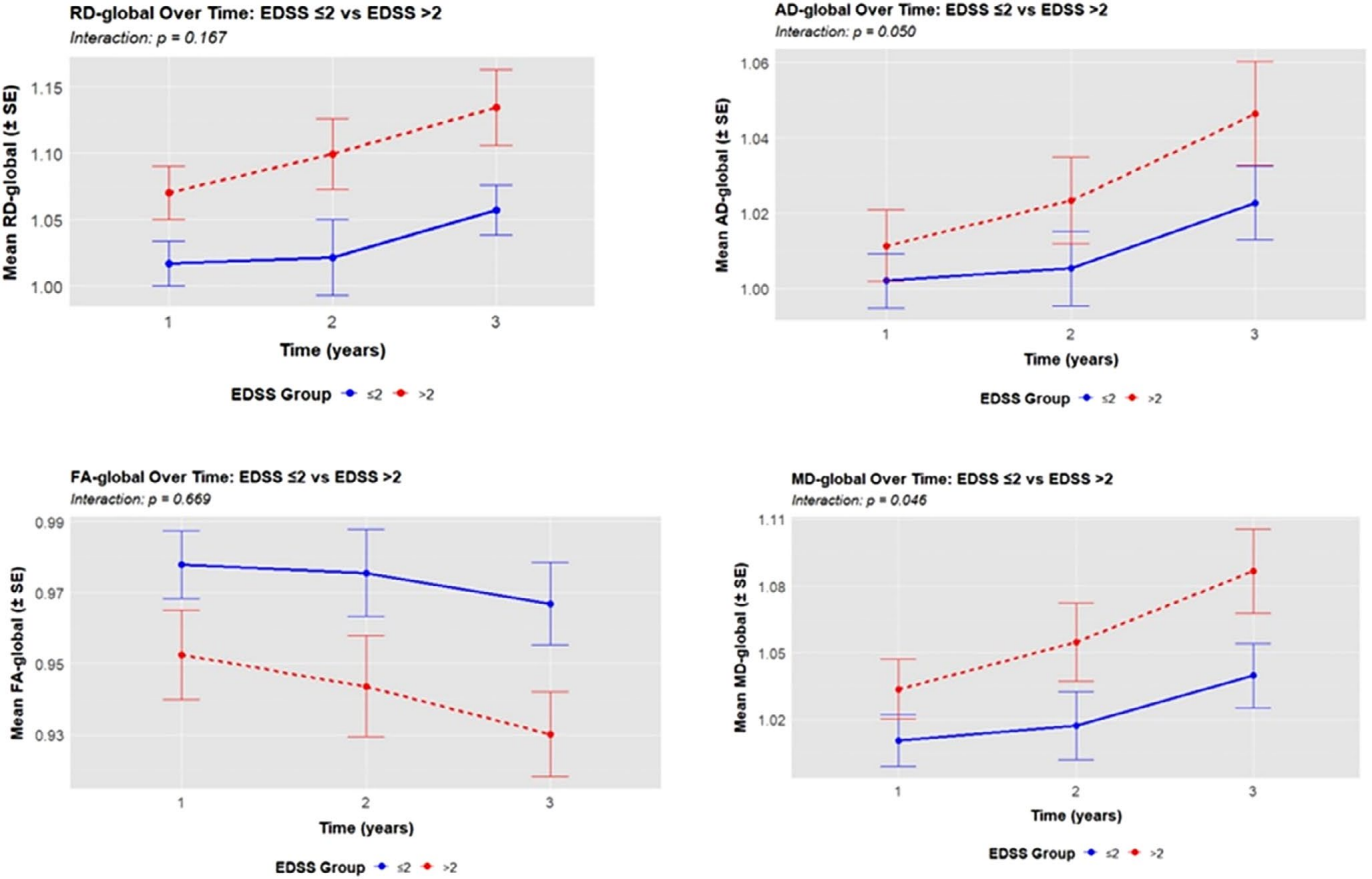
Longitudinal comparison of qMRI metrics (RD, AD, MD, FA) slopes between EDSS ≤ 2 and EDSS > 2 groups, showing changes from baseline (V0) to follow‐up (V1, V2). AD, axial diffusivity; EDSS, expanded disability status scale; FA, fractional anisotropy; MD, mean diffusivity; RD, radial diffusivity; SE, standard error.

For RD‐global, a significant increase was seen in both EDSS subgroups (*p* < 0.001), with a large effect size for both groups (*η* ^2^G = 0.77–0.78). In the EDSS ≤ 2 group, there was a statistically significant increase in mean RD of 0.043 (SE = 0.015) between baseline and Visit 2. The mean increase in the EDSS > 2 group between baseline and Visit 2 was 0.083 (SE = 0.015). However, when the slopes of increase were compared between the two groups, there was no significant difference in the increase of RD (Figure 3).

For AD‐global, significant increases were observed across baseline and V2 for both subgroups (*p* = 0.042–0.001), with large effect sizes (*η* ^2^G = 0.55–0.91). The EDSS ≤ 2 group showed an increase of 0.021 (SE = 0.009) from 1.002 (SE = 0.009) at V0 to 1.023 (SE = 0.009) at V2, while the EDSS > 2 group showed an increase of 0.043 (SE = 0.009) at V2. The increase in slopes of the two groups was borderline not significant (*p* = 0.050) (Figure 3).

For MD‐global, a significant increase was observed in both subgroups (*p* = 0.019–0.001), with effect sizes ranging from η^2^G = 0.57–0.84. The EDSS ≤ 2 group showed an increase of 0.030 (SE = 0.011) between V0 and V2, while the EDSS > 2 group showed a more substantial increase of 0.062 (SE = 0.012) between V0 and V2. When the slopes of both groups were compared, there was a statistically significant difference (*p* = 0.046) (Figure 3).

For FA‐global, significant reductions were observed over time (*p* = 0.007–0.003), with moderate‐to‐large effect sizes (η^2^G = 0.53–0.71) for both subgroups. In the EDSS ≤ 2 group, FA‐global decreased with a mean difference of −0.17 (SE = 0.007) between V2 and V0. The EDSS > 2 group showed a greater decline of −0.20 (SE = 0.006) between V2 and V0. However, there were no statistically significant changes in the slopes of both subgroups (*p* = 0.669) (Figure 3). See Table 5 for further detailed results.

**TABLE 5:** Longitudinal changes in qMRI parameters (RD, AD, MD, FA) by EDSS subgroups, with estimated marginal means (V0, V1, V2) and effect sizes (*η* ^2^).

qMRI parameter	EDSS subgroup	*N*	V0	V1	V2	*p* *	Effect size**	V2 vs. V0
RD‐global	EDSS ≤ 2	15	1.022 (0.020)	1.040 (0.020)	1.066 (0.020)	< 0.001	0.78	0.043 (0.015)
	EDSS > 2	10	1.040 (0.020)	1.083 (0.023)	1.131 (0.22)	< 0.001	0.77	0.083 (0.015)
AD‐global	EDSS ≤ 2	15	1.002 (0.009)	1.005 (0.009)	1.023 (0.009)	< 0.001	0.55	0.021 (0.009)
	EDSS > 2	10	1.011 (0.011)	1.023 (0.011)	1.046 (0.011)	0.042	0.91	0.043 (0.009)
MD‐global	EDSS ≤ 2	15	1.011 (0.013)	1.024 (0.014)	1.043 (0.014)	0.019	0.57	0.030 (0.011)
	EDSS > 2	10	1.025 (0.016)	1.049 (0.016)	1.086 (0.016)	< 0.001	0.84	0.062 (0.012)
FA‐global	EDSS ≤ 2	15	0.979 (0.011)	0.973 (0.011)	0.962 (0.011)	0.014	0.53	−0.17 (0.007)
	EDSS > 2	10	0.954 (0.012)	0.946 (0.012)	0.934 (0.012)	0.007	0.71	−0.20 (0.006)

Abbreviations: AD, axial diffusivity; EDSS, expanded disability status scale; effect size, partial eta squared for the repeated measures analyses of variance; FA, fractional anisotropy displayed as estimated marginal means (SE); MD, mean diffusivity; RD, radial diffusivity; V0, estimated marginal means for baseline; V1, estimated marginal means for follow‐up year 1; V2 vs. V0, significant mean difference between qMRI value follow‐up year 2 vs. baseline pairwise comparison (SE); V2, estimated marginal means for follow‐up year 2.

* Bonferroni corrected *p* value for post hoc pairwise comparisons.

** Partial eta squared for the repeated measures analyses of variance.

## 4 Discussion

In this study, we aimed to assess the utility of brainQuant‐derived quantitative MRI (qMRI) parameters as a generalizable, fully automatized tool for both early detection of cerebral ALD (CALD) lesions and monitoring myelopathy progression in adult males with X‐linked adrenoleukodystrophy (X‐ALD).

Our results show that while DTI metrics do not differ between X‐ALD patients with and without CALD, at baseline, they demonstrated sensitivity to longitudinal changes associated with myelopathy.

### 4.1 CALD

We found no statistically significant differences in DTI parameters between patients with and without CALD in our baseline analyses. When analyzing individual brain regions commonly affected in CALD, only the splenium of the corpus callosum showed a marginally significant increase in RD, which did not persist after correction for multiple comparisons. These findings suggest that the brainQuant module, in its current implementation, was not able to reliably differentiate between CALD and normal appearing white matter (NAWM) in non‐CALD patients.

Previous studies that showed differences in qMRI parameters in NAWM and CALD lesions used different approaches.

Van der Voorn et al. conducted ROI analyses of qMRI markers (Apparent diffusion coefficient (ADC), FA, Magnetization Transfer Ratio (MTR), and Magnetic Resonance Spectroscopy (MRS)) in living and post‐mortem X‐ALD patients, comparing NAWM, lesion edges, and lesion cores [27]. Differences were seen between abnormal white matter regions in CALD patients and NAWM within CALD patients [27]. Unlike our study, their focus was mainly on the differences in white matter abnormalities in CALD patients and differences between CALD and healthy controls, rather than comparing CALD to non‐CALD NAWM. Similarly, another study [28] focusing on microstructural alterations between lesions and NAWM within CALD patients showed differences in qMRI parameters (ADC, FA). But they also did not compare CALD to non‐CALD X‐ALD patients or controls.

Contrary to our findings, recent work by Pierpont et al. [12] evaluated the use of diffusion tensor imaging (DTI) to assess microstructural white matter integrity in boys with ALD. Similar to our study design, they compared ALD patients with cerebral involvement to those without, rather than comparing to external healthy controls. Their study demonstrated that, even in patients with mild posterior lesions (Loes 1–2), there were measurable changes in FA, AD, and particularly in the splenium of the corpus callosum, compared to patients with no cerebral lesions.

Therefore, it is surprising that CALD did not differ from non‐CALD patients in our study. This lack of significant differentiation may be attributed to the small sample size of CALD patients within our cohort, and it has to be kept in mind that the difference in the group sizes (*n* = 15 vs. *n* = 47) poses difficulties for comparison. Additionally, the heterogeneity in lesion distribution and disease severity among CALD patients could contribute to the observed lack of significant differences.

We applied a clinically employable qMRI approach using the brainQuant module, which expresses qMRI values as standardized deviations from normative control values and averages measurements across multiple white matter regions. This atlas‐based method, focusing on global diffusion changes, contrasts with Pierpont et al.'s localized DTI analysis of NAWM [12]. The limitation of this global approach is that it may dilute focal pathological signals. This is vividly illustrated by the case of patient XALD_007 (Figure S4). While the group analysis showed no significance, this individual CALD patient exhibited severe, focal increases in RD (> 3 SD) specifically in classic CALD regions like the splenium of the corpus callosum and parietal white matter (Figure S5), with clear progression over time (Figure S6). This case confirms that the pathology is detectable but highlights that our group‐level analysis, which averaged values across large tracts, was insufficiently sensitive to these focal changes.

Our findings underscore the limitations of pragmatic qMRI for detecting microstructural distinctions in X‐ALD, suggesting a need for more sensitive, localized approaches.

### 4.2 Myeloneuropathy

In this study, we categorized patients into EDSS ≤ 2 (mild disability, 0–2) and EDSS > 2 (more than mild disability, 2.5 and above) groups to distinguish minimal from significant impairment. Patients with EDSS ≤ 2 exhibit no symptoms to minimal disability, remaining fully ambulatory with subtle, manageable symptoms that rarely cause complaints. In contrast, EDSS > 2 reflects moderate disability (e.g., noticeable gait disturbance at 2.5) to significant impairment. The EDSS: 2.0 cutoff marks a clinical transition from mild to more affected states, where disability begins to disrupt daily life [29]. Patients with an EDSS score > 2.0 exhibited significantly higher RD‐global, MD‐global, and AD‐global values, while FA‐global was lower, consistent with previous findings in neurodegenerative myelopathies [30, 31, 32]. When looking at individual regions, RD was significantly different in anatomic regions such as the corpus callosum, cerebral peduncles, internal capsule, and superior longitudinal fasciculus. These regional changes correlated strongly with clinical outcome measures, including the EDSS, SSPROM, and 6‐MWT. These findings are consistent with previous studies on DTI metrics and spinal cord involvement, which showed that qMRI parameters in the spinal cord and cerebral corticospinal tracts can reveal structural abnormalities in white matter tracts that are not detectable by conventional imaging [15, 33], and correlate with clinical disability and reveal early structural changes even in asymptomatic individuals. In our study, RD‐global and AD‐global showed significant increases over time in both EDSS subgroups, with large effect sizes. However, the rate of progression did not differ significantly between the EDSS ≤ 2.0 and EDSS > 2.0 groups, indicating that myeloneuropathy progression follows a similar course across different disease severities. Notably, the increase in RD‐global was particularly robust, supporting its potential as a biomarker for disease progression. Conventional clinical outcome measures like the EDSS, SSPROM, and 6‐MWT are not sensitive enough to detect early or subtle disease progression [8, 14]. Yska et al. showed in a recent publication that patients with an EDSS ≤ 2.5 showed no measurable clinical decline over short follow‐up durations detected by the currently used outcome measures such as the 6‐MWT, whereas patients with an EDSS > 2.5 showed more consistent progression. This discrepancy emphasizes the need for surrogate outcome measures that overcome the limitations of current clinical tools. The slow progression of myelopathy in ALD makes it impractical to rely solely on functional scales in therapeutic trials, especially those involving presymptomatic individuals. Our findings suggest that global qMRI metrics, which are standardized, reproducible, and less susceptible to motivational or measurement bias, can fill this gap.

In conclusion, our results suggest that while generalized diffusion MRI parameters were not effective in distinguishing between CALD and non‐CALD patients, they demonstrated potential for monitoring myelopathy progression over time. The ability to track disease progression consistently across different patient subgroups highlights the utility of qMRI as a valuable tool for clinical trials and longitudinal disease monitoring in X‐ALD.

## Author Contributions

**Eda G. Kabak:** data curation, formal analysis, investigation, writing – original draft. **Fenna van Dodewaard:** data curation, formal analysis. **Stefan D. Roosendaal:** data curation, validation, writing – review and editing. **Hemmo A. F. Yska:** data curation, writing – review and editing. **Marije M. C. Voermans:** data curation, project administration. **Marine Bourgeais:** writing – review and editing. **Vincent Perlbarg:** methodology, software, writing – review and editing. **Marc Engelen:** conceptualization, supervision, funding acquisition, writing – review and editing; Guarantor author.

## Conflicts of Interest

The brainTale‐care platform and its brainQuant module were provided by brainTale without any form of financial compensation for the purposes of this research for AUMC researchers. The AUMC‐related authors did not receive any form of financial compensation, funding, or other material support from brainTale or any related entity in connection with the preparation, authorship, or publication of this article. V. Perlbarg provided background information on the pipeline processing of the brainQuant module. He is a co‐founder and employee of BrainTale, holds stock options in the company, and is a named inventor on related patents. M. Bourgeais is employee of BrainTale. M. Engelen: is co‐PI for Spur Therapeutics (CYGNET andPROPEL trials) and was co‐PI for Minoryx (ADVANCE trial). He has received research support from Minoryx, Autobahn Therapeutics, BlueBirdBio and SwanBio (now Spur Therapeutics); consultation fees from Minoryx, BlueBirdBio, Autobahn Therapeutics and Poxel. Other authors report no further disclosures.

## Supporting information


**Figure S1:** Box‐plot RD‐Global Cerebral ALD vs. No Cerebral ALD patients.

**Figure S2:** Between group differences qMRI parameters EDSS subgroups.

**Figure S3:** Correlation RD‐global and clinical parameters.

**Figure S4:** Correlation RD‐global and clinical parameters.

**Figure S5:** Correlation RD‐global and clinical parameters.

**Figure S6:** Correlation RD‐global and clinical parameters.

**Table S1:** Between group differences individual regions: cALD versus no cALD.
**Table S2:** Between group differences individual regions: EDSS ≤ 2 and EDSS > 2.
**Table S3:** Correlation coefficients RD‐individual anatomic regions and clinical outcome measures.

## Data Availability

The data that support the findings of this study are available on request from the corresponding author. The data are not publicly available due to privacy or ethical restrictions.
