# Supplementary figures and images for: An Automated Analysis Tool for Diffusion Tensor Imaging‐Based Quantitative MRI in X‐Linked Adrenoleukodystrophy
Source: J Inherit Metab Dis. 2025 Oct 26;48(6):e70108. doi: 10.1002/jimd.70108 (PMC12555024; doi:10.1002/jimd.70108)

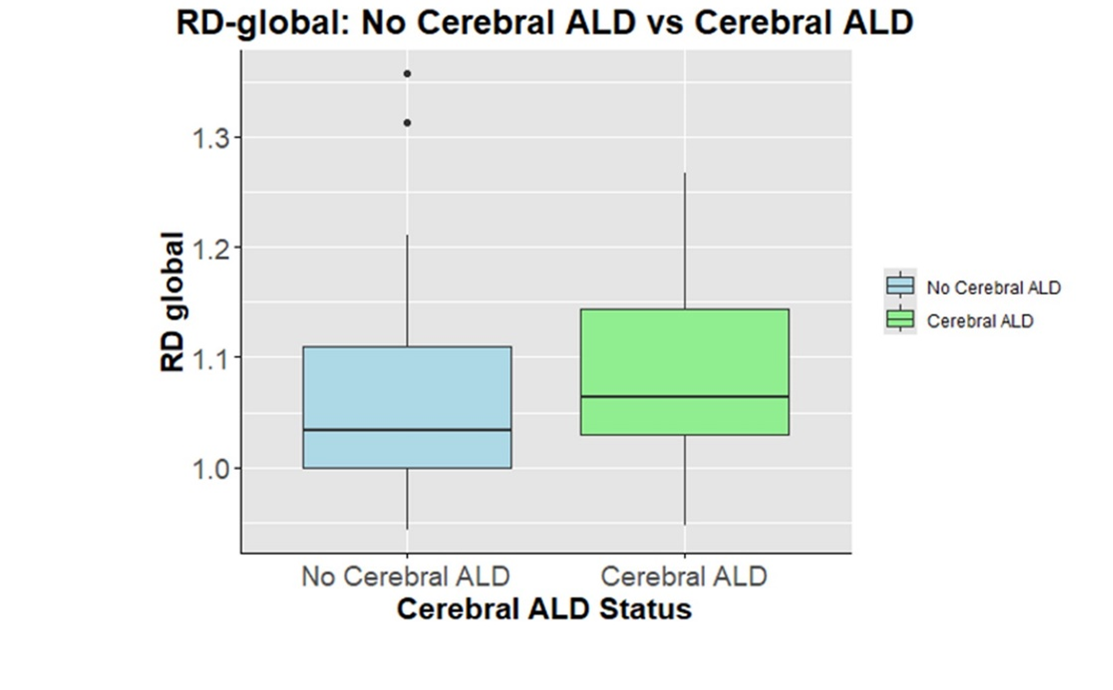

Supplement: Supplementary file 1 — Figure S1: Box‐plot RD‐Global Cerebral ALD vs. No Cerebral ALD patients. [file JIMD-48-0-s003.tif]

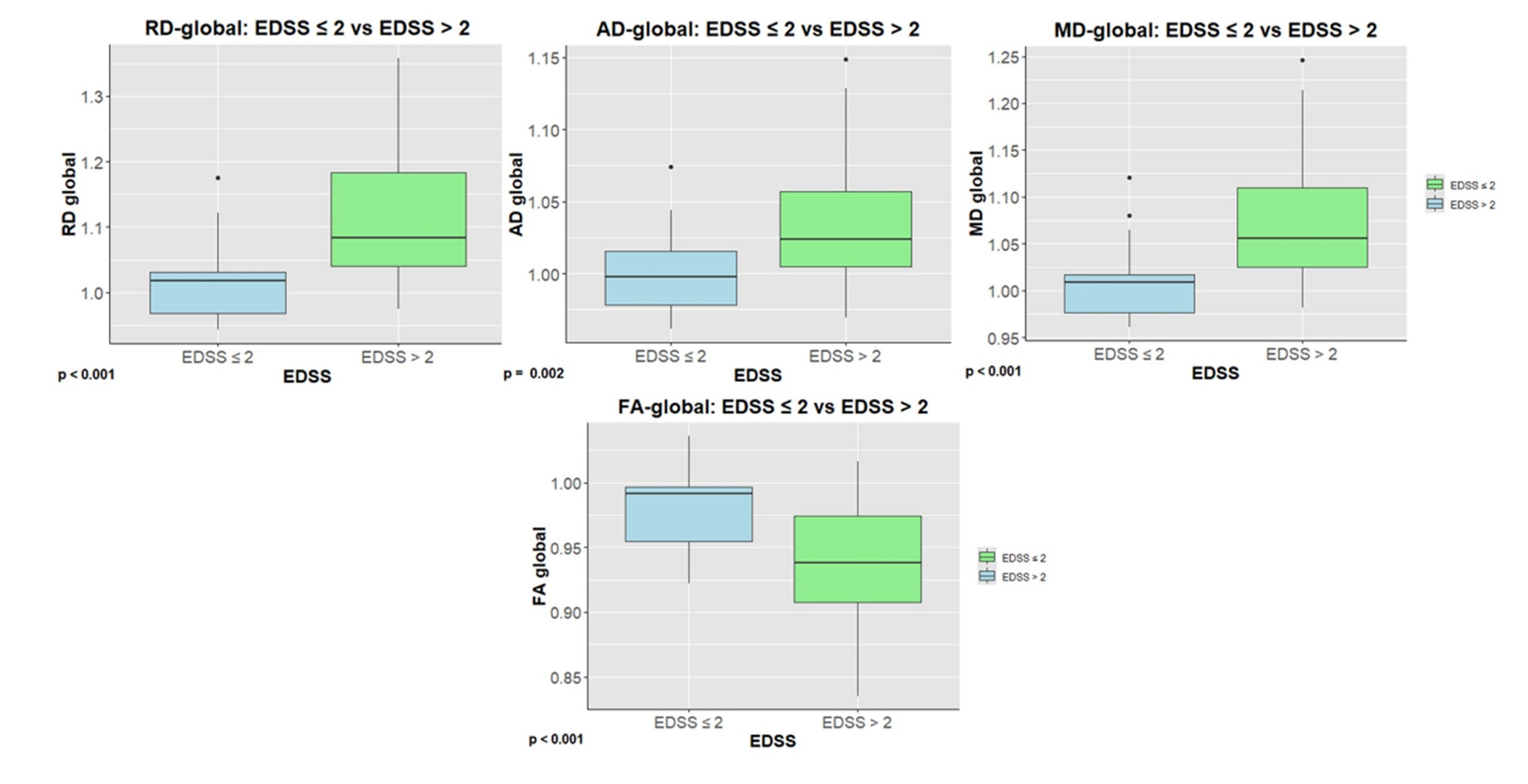

Supplement: Supplementary file 2 — Figure S2: Between group differences qMRI parameters EDSS subgroups. [file JIMD-48-0-s006.tif]

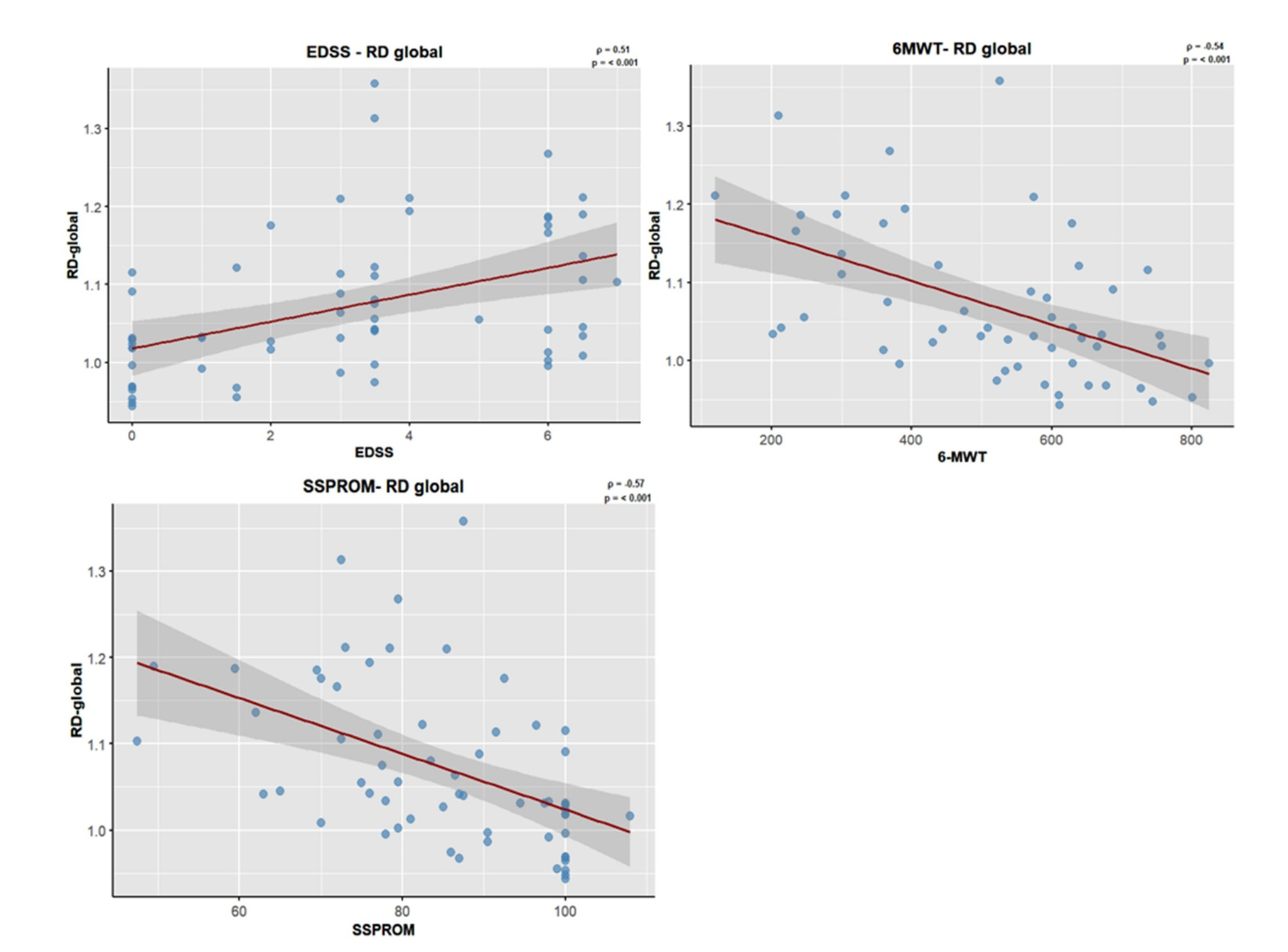

Supplement: Supplementary file 3 — Figure S3: Correlation RD‐global and clinical parameters. [file JIMD-48-0-s007.tif]

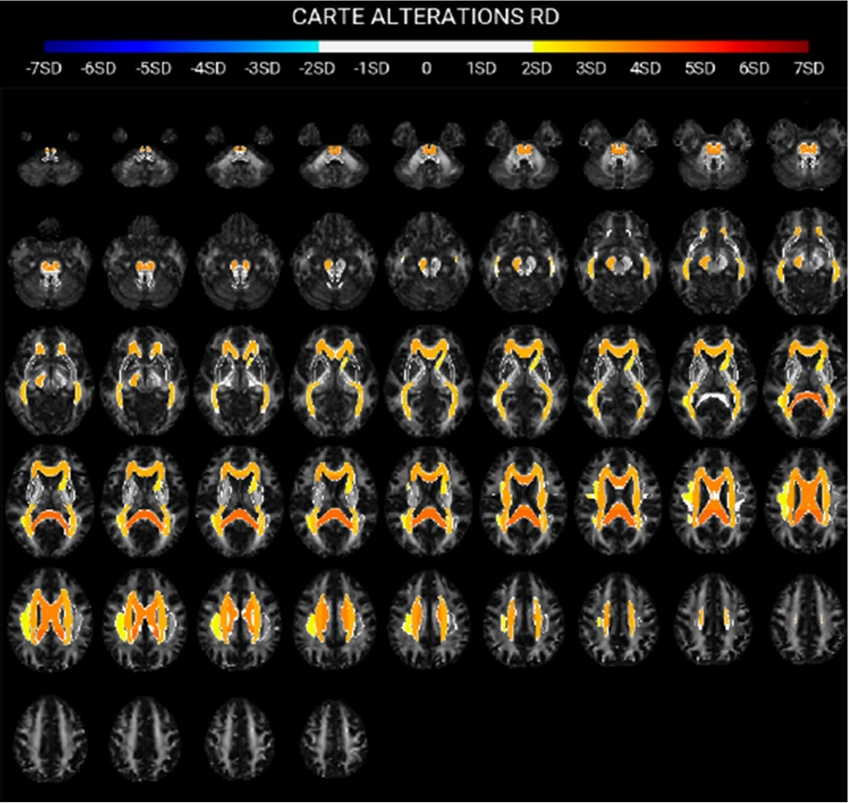

Supplement: Supplementary file 4 — Figure S4: Correlation RD‐global and clinical parameters. [file JIMD-48-0-s002.tif]

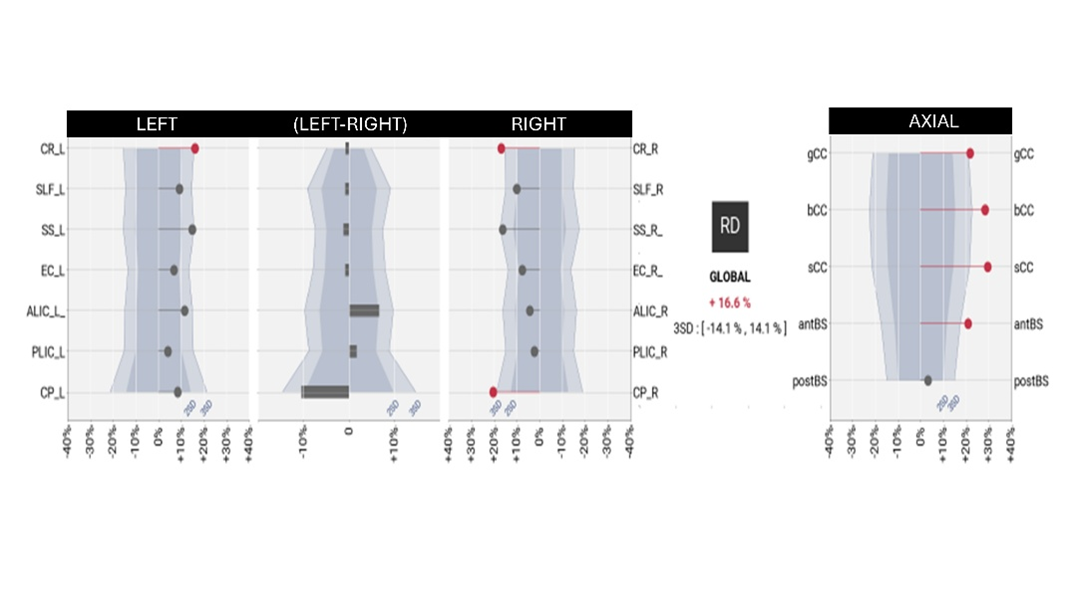

Supplement: Supplementary file 5 — Figure S5: Correlation RD‐global and clinical parameters. [file JIMD-48-0-s005.tif]

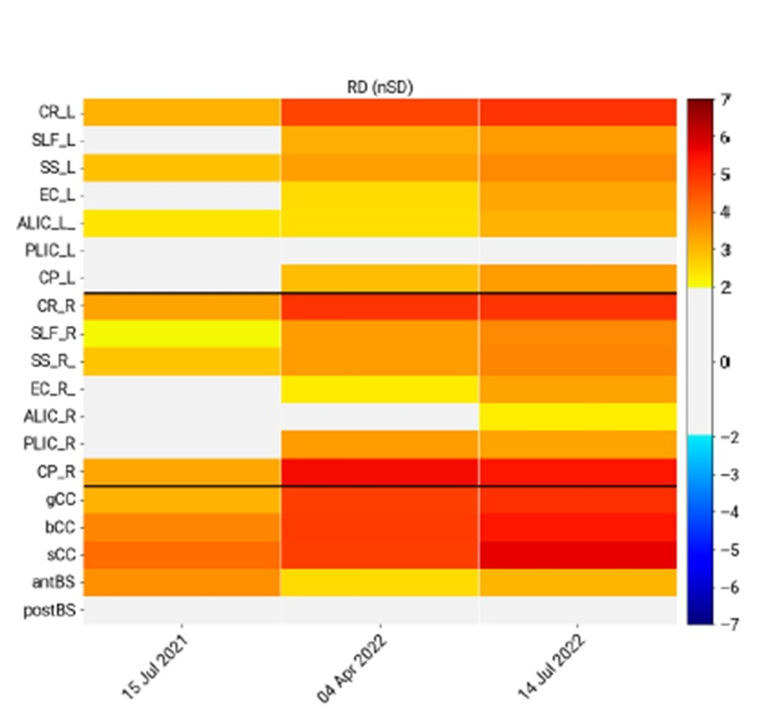

Supplement: Supplementary file 6 — Figure S6: Correlation RD‐global and clinical parameters. [file JIMD-48-0-s004.tif]
