# Supplementary material for: An Automated Analysis Tool for Diffusion Tensor Imaging‐Based Quantitative MRI in X‐Linked Adrenoleukodystrophy
Source: J Inherit Metab Dis. 2025 Oct 26;48(6):e70108. doi: 10.1002/jimd.70108 (PMC12555024; doi:10.1002/jimd.70108)
Supplement: Supplementary file 7 — Table S1: Between group differences individual regions: cALD versus no cALD. Table S2: Between group differences individual regions: EDSS ≤ 2 and EDSS > 2. Table S3: Correlation coefficients RD‐individual anatomic regions and clinical outcome measures. [file JIMD-48-0-s001.docx]

# Appendix

| **qMRI: Individual regions** | **No cALD** | **cALD** | **p-value*** | **adjusted p-value**** |
| --- | --- | --- | --- | --- |
| **RD - antBS Anterior brainstem** | 31.23 | 32.33 | 0.845 | 1.000 |
| **RD - postBS Posterior brainstem** | 30.13 | 35.80 | 0.296 | 1.000 |
| **RD - Right cerebral peduncle** | 30.44 | 34.80 | 0.424 | 1.000 |
| **RD - Left cerebral peduncle** | 31.40 | 31.80 | 0.948 | 1.000 |
| **RD - Genu of the corpus callosum** | 32.77 | 27.53 | 0.335 | 1.000 |
| **RD - Body of the corpus callosum** | 30.83 | 33.60 | 0.614 | 1.000 |
| **RD - Splenium of the corpus callosum** | **28.81** | **39.93** | **0.037** | **0.748** |
| **RD - Anterior arm of the internal capsule (right)** | 31.13 | 32.67 | 0.782 | 1.000 |
| **RD - Anterior arm of the internal capsule (left)** | 31.00 | 33.07 | 0.708 | 1.000 |
| **RD - Posterior arm of the internal capsule (right)** | 30.98 | 33.13 | 0.696 | 1.000 |
| **RD - Posterior arm of the internal capsule (left)** | 30.94 | 33.27 | 0.672 | 1.000 |
| **RD - Sagittal stratum (right)** | 30.38 | 35.00 | 0.397 | 1.000 |
| **RD - Sagittal stratum (left)** | 31.68 | 30.93 | 0.897 | 1.000 |
| **RD - Superior longitudinal fasciculus (right)** | 32.17 | 29.40 | 0.614 | 1.000 |
| **RD - Superior longitudinal fasciculus (left)** | 31.57 | 31.27 | 0.961 | 1.000 |
| **RD - External capsule (right)** | 31.09 | 32.80 | 0.757 | 1.000 |
| **RD - External capsule (left)** | 31.64 | 31.07 | 0.922 | 1.000 |
| **RD - Corona radiata (right)** | 30.77 | 33.80 | 0.580 | 1.000 |
| **RD - Corona radiata (left)** | 31.26 | 32.27 | 0.856 | 1.000 |
| ***Mann-Whitney U test, ** Bonferroni adjusted significance, Abbreviations: RD (Radial Diffusivity)** | | | |  |

***Supplementary Table 1: Between group differences individual regions: cALD vs no cALD***

RD Radial Diffusivity

cALD Cerebral Adrenoleukodystrophy

p-value: Mann-Whitney U test

adjusted p-value: Bonferroni correction for multiple comparisons

| **qMRI: Individual regions** | **EDSS ≤ 2.0** | **EDSS >2** | **p-value*** | **adjusted p-value**** |
| --- | --- | --- | --- | --- |
| **RD - antBS Anterior brainstem** | 23.96 | 36.26 | **<0.001** | 0.168 |
| **RD - postBS Posterior brainstem** | 24.58 | 35.87 | **<0.001** | 0.319 |
| **RD - Right cerebral peduncle** | **21.58** | **37.76** | **<0.001** | **0.009** |
| **RD - Left cerebral peduncle** | **19.88** | **38.84** | **<0.001** | **0.001** |
| **RD - Splenium of the corpus callosum** | 24.92 | 35.66 | **<0.001** | 0.441 |
| **RD - Anterior arm of the internal capsule (right)** | 25.38 | 35.37 | **<0.001** | 0.670 |
| **RD - Anterior arm of the internal capsule (left)** | 23.58 | 36.50 | **<0.001** | 0.111 |
| **RD - Posterior arm of the internal capsule (right)** | 23.75 | 36.39 | **<0.001** | 0.134 |
| **RD - Posterior arm of the internal capsule (left)** | **21.17** | **38.03** | **<0.001** | **0.005** |
| **RD - External capsule (right)** | **21.79** | **37.63** | **<0.001** | **0.012** |
| **RD - External capsule (left)** | **21.58** | **37.76** | **<0.001** | **0.009** |
| **RD - Corona radiata (right)** | **21.50** | **37.82** | **<0.001** | **0.008** |
| **RD - Corona radiata (left)** | **21.77** | **37.64** | **<0.001** | **0.015** |
| ***Mann-Whitney U test, ** Bonferroni adjusted p-value for multiple comparisons, Abbreviations: qMRI (quantitative MRI), RD (Radial Diffusivity), EDSS (Expanded Disability Status Scale)** | | | | |

***Supplementary Table 2: Between group differences individual regions: EDSS ≤2 and EDSS >2***

RD Radial Diffusivity

cALD Cerebral Adrenoleukodystrophy

p-value: Mann-Whitney U test

adjusted p-value: Bonferroni correction for multiple comparisons

| **RD- Specific anatomic regions Spearn’s rho** | **EDSS** | | | **SSPROM** | | | **6MWT** | | |
| --- | --- | --- | --- | --- | --- | --- | --- | --- | --- |
|  | Correlation coefficient | *p-*value* | N | Correlation coefficient | p-value* | N | Correlation coefficient | p-value* | N |
| **Genu corpus callosum** | 0.51 | <0.001 | 62 | -0.49 | <0.001 | 62 | -0.48 | <0.001 | 54 |
| **Body corpus callosum** | 0.49 | <0.001 | 62 | -0.51 | <0.001 | 62 | -0.46 | <0.001 | 54 |
| **Splenium corpus callosum** | 0.25 | 0.050 | 62 | -0.33 | 0.01 | 62 | -0.36 | 0.007 | 54 |
| **Right ALIC** | 0.33 | 0.009 | 62 | -0.32 | 0.01 | 62 | -0.43 | 0.001 | 54 |
| **Left ALIC** | 0.36 | 0.004 | 62 | -0.39 | 0.002 | 62 | -0.43 | 0.001 | 54 |
| **Right PLIC** | 0.34 | 0.007 | 62 | -0.37 | 0.003 | 62 | -0.43 | 0.001 | 54 |
| **Left PLIC** | 0.38 | 0.007 | 62 | -0.43 | <0.001 | 62 | -0.43 | 0.001 | 54 |
| **Anterior brainstem** | 0.25 | 0.050 | 62 | -0.32 | 0.011 | 62 | -0.32 | 0.02 | 54 |
| **Posterior brainstem** | 0.35 | 0.005 | 62 | 0.46 | <0.01 | 62 | -0.24 | 0.085 | 54 |
| **Abbreviations: ALIC (Anterior limb of internal capsule), PLIC (Posterior limb of internal capsule) *p-value: after Bonferroni correction for multiple comparisons** | | | | | | | | | |

***Supplementary Table 3: Correlation coefficients RD- individual anatomic regions and clinical outcome measures***

SSPROM Severity Score for Progressive Myelopathy

EDSS Expanded Disability Status Scale

6-MWT 6-minutes walking test

ALIC Anterior Limb of Internal Capsule

PLIC Posterior Limb of Internal Capsule

Correlation coefficient: Spearman rank order/ Pearson correlation

p-value:Bonferroni correction for multiple comparisons
